# MiR-205 as a promising biomarker in the diagnosis and prognosis of lung cancer

**DOI:** 10.18632/oncotarget.20262

**Published:** 2017-08-14

**Authors:** Jing-Hua Li, Shan-Shan Sun, Ning Li, Peng Lv, Shu-Yang Xie, Ping-Yu Wang

**Affiliations:** ^1^ Department of Epidemiology, Binzhou Medical University, YanTai, ShanDong 264003, P.R. China; ^2^ Key Laboratory of Tumor Molecular Biology in Binzhou Medical University, Department of Biochemistry and Molecular Biology, Binzhou Medical University, YanTai, ShanDong 264003, P.R. China

**Keywords:** biomarker, miR-205, lung cancer, diagnosis, prognosis

## Abstract

MicroRNA-205 (miR-205) was revealed as a novel diagnostic and prognostic biomarker for lung cancer, but the results in the published papers were inconsistent. This meta-analysis aimed to investigate the diagnostic and prognostic roles of miR-205 in patients with lung cancer. Totally, 16 eligible articles were included, among which 10 articles investigated the diagnostic value of miR-205, 5 articles examined its prognostic values, and 1 article studied both diagnostic and prognostic values. For the diagnostic meta-analysis, the pooled sensitivity, specificity, positive likelihood ratio, negative likelihood ratio, diagnostic odds ratio, and the overall area under the curve of miR-205 for patients with lung cancer were 0.88 (95% *CI* = 0.78 – 0.94), 0.78 (95% *CI* = 0.66 – 0.86), 4.00 (95% *CI* = 2.47 – 6.49), 0.16 (95% *CI* = 0.08 – 0.30), 25.86 (95% *CI* = 9.29 – 71.95), and 0.90 (95% *CI* = 0.87 – 0.92), respectively, indicating that miR-205 is a useful biomarker for diagnostic of lung cancer. The subgroup analysis further demonstrated that miR-205 had an excellent overall accuracy for detection with tissue samples compare with blood samples. For the prognostic meta-analysis, the pooled outcome of the disease-free survival and recurrence-free survival analyses revealed that increased miR-205 levels had a protective role in the prognosis of patients with lung cancer (pooled HR = 0.86, 95% CI: 0.78-0.96, z = 2.83, *P* = 0.005). In conclusion, miR-205 may be a promising biomarker for detection, predicting the recurrence of patients with lung cancer.

## INTRODUCTION

Lung cancer is the leading cause of cancer-related death among men and women worldwide with only a low 15% overall 5-year survival rate and a high recurrence rate [1–5]. Approximately 85% of all lung cancers are non-small cell lung cancer (NSCLC), which represents heterogeneous subtypes including most of squamous cell lung carcinoma (SCC), adenocarcinoma (ADC), and large-cell lung carcinoma (SCLC) [6]. Early detection and classification of NSCLC needed to be extended by extensive molecular studies [7]. Considering the increasing gene targets of cancer therapy and their limits in clinical application [7, 8], a precise molecular biomarker for early detection, accurate assessment, personalizing therapy, and prognosis evaluation for lung cancer needed to be explored [9].

MicroRNAs (miRNAs), as endogenous and non-coding small RNAs, suppress gene expression by binding to 3′- untranslated region (UTR) of targeted messenger RNAs (mRNAs), leading to gene degradation or translation suppression [10, 11]. Dysregulation of miRNAs plays crucial roles in lung cancer development, progression, and response to therapy [12–14]. Emerging evidence suggests that miRNAs might be predominant diagnostic and prognostic biomarkers for lung cancer [15–17].

MiR-205, located in a lung cancer-associated genomic amplification region at 1q32.2 [18, 19], participates in tumorigenesis of lung cancer [20, 21], especially in the occurrence, development, and prognosis of NSCLC [22, 23]. It markedly overexpresses in the tissues of lung cancer and serves as a prospective diagnostic biomarker for pulmonary diseases. Overexpression of miR-205 promoted NSCLC cell invasion and metastasis through regulating an epithelial phenotype with increased E-cadherin and reduced fibronectin [22]. MiR-205 was further developed to identify SCC and ADC subtypes of NSCLC [23]. These studies indicated that miR-205 might serve as a potential biomarker for detection of NSCLC. Meanwhile, substantial evidence reveals that level of miR-205 was related to the prognosis of lung cancer [24–29]. However, the results in these studies were inconsistent and remained inconclusive. Therefore, we conducted this meta-analysis to evaluate the diagnostic and prognostic efficiency of miR-205 for patients with lung cancer.

## RESULTS

### Literature selection

**Figure 1:**
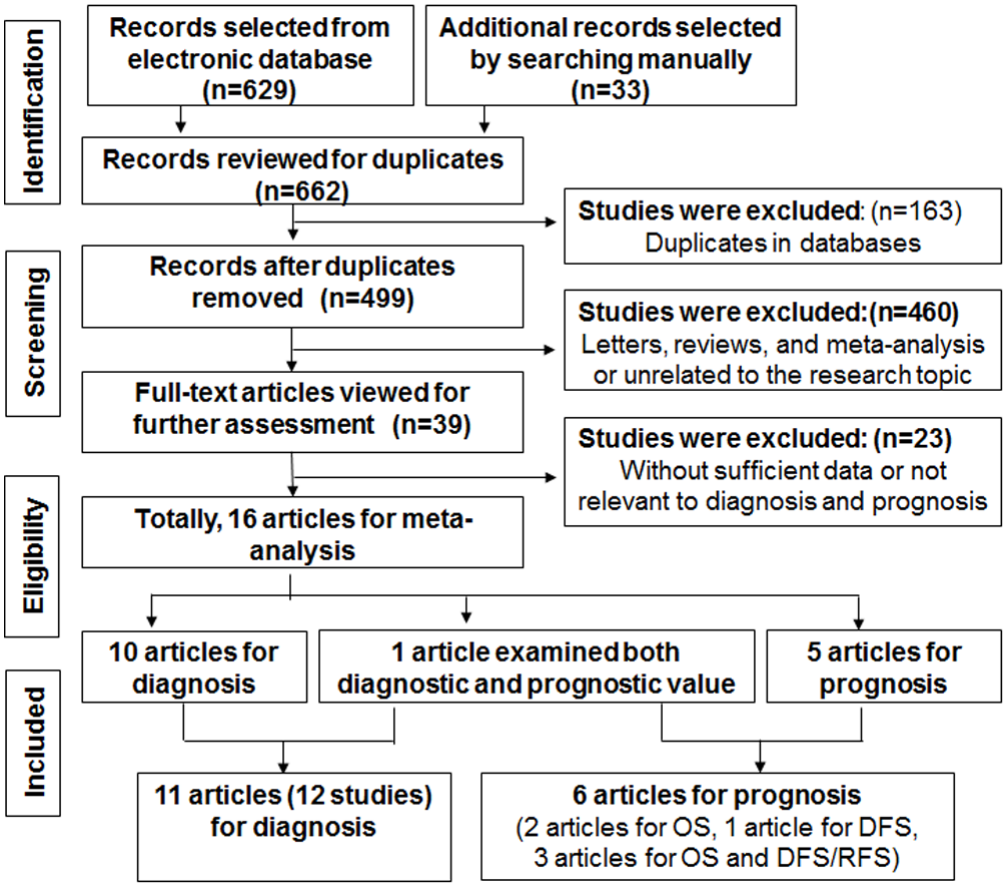
Flow diagram of study selection process

A total of 662 potentially relevant articles were obtained from electronic databases and other sources, and 499 of them were remained after removing 163 duplications. Of the remained articles, 460 articles, such as letters, reviews and meta-analysis, or unrelated to the research topic, were excluded after reviewing titles and abstracts. Of the remaining 39 full-text candidates, 23 potential articles were further excluded, which did not have sufficient data, or were unrelated to cancer diagnosis or prognosis. Finally, 16 eligible articles [18, 23–37] were included in this study (Figure 1). Among the included 16 articles, 1 article examined both diagnostic and prognostic values of miR-205. Thus, 11 articles reported the diagnostic values of miR-205, and 6 articles examined prognostic values of miR-205.

### Diagnostic meta-analysis

#### Study characteristics and quality assessment

**Figure 2:**
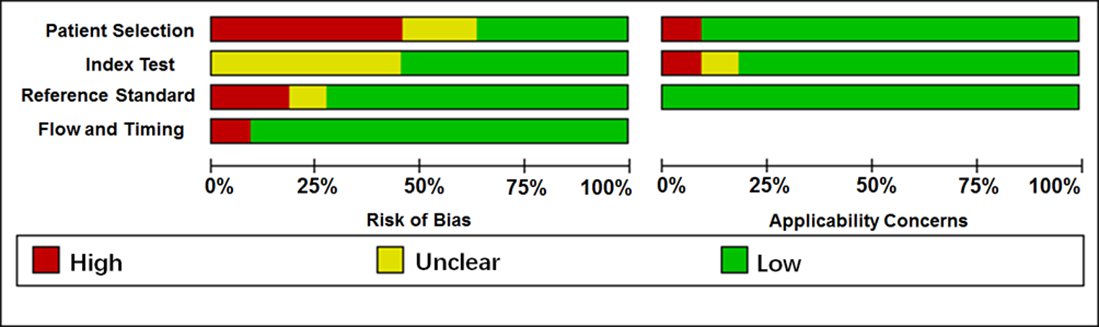
Quality assessment of diagnostic accuracy for the included studies

Totally, 564 lung cancer patients (mainly composed of SCC patients) and 667 controls (with healthy people and non-SCC patients) were included in this meta-analysis. Because Hamamoto et al [32] investigated the roles of miR-205 in two independent study samples, we considered it as two independent studies in this analysis. Thus, totally 11 articles including 12 studies were included (Table 1). The investigated ethnicity included Asian (n = 4), Caucasian (n = 6), and Caucasian/African (n = 2). The specimen types contained tissue (n = 7) and blood (n = 5). The qualities of the included studies were assessed by Quality Assessment of Diagnostic Accuracy Studies-2 (QUADAS-2, Supplementary Figure 1), and each study received moderate or high quality with scores between 4 and 6. The QUADAS-2 results are represented in Figure 2 and Table 1, showing that no significant bias was presented in current meta-analysis.

**Table 1:** Characteristics and quality assessment of diagnostic clinical trials included in the meta-analysis

Author	Year	Country	Ethnicity	Cancer type	Case/control	Specimen	AUC	TP	FP	FN	TN	SEN	SPE	QUADAS
Lebanony	2009	USA	Caucasian	SCC	24/49	tissue	0.960	23	5	1	44	0.96	0.90	5
Xing	2010	USA	Caucasian /African	SCC	48/48	blood	0.789	31	5	17	43	0.65	0.90	5
Del	2011	Italy	Caucasian	SCC	24/26	tissue	NM	24	5	0	21	1.00	0.81	4
Le	2012	China	Asian	Lung cancer	82/50	blood	0.810	70	14	12	36	0.85	0.72	5
Hamamoto	2013	Japan	Asian	SCC	25/54	tissue	NM	19	20	6	34	0.76	0.63	6
				SCC	44/44	tissue	NM	38	17	6	27	0.86	0.62	
Molina-Pinelo	2014	Spain	Caucasian	SCC	25/19	tissue	NM	25	5	0	14	1.00	0.76	4
Shen	2014	USA	Caucasian /African	Lung cancer	66/68	blood	0.620	36	26	30	42	0.55	0.62	6
Huang	2014	China	Asian	SCC	45/152	tissue	0.983	43	3	2	149	0.96	0.98	6
Patnaik	2015	USA	Caucasian	SCC	28/49	tissue	0.910	23	9	5	40	0.82	0.82	5
Halvorsen	2016	Norway	Caucasian	Lung cancer	100/58	blood	0.800	93	32	7	26	0.93	0.44	6
Zaporozhchenko	2016	Russia	Caucasian	SCC	53/50	serum	0.684	41	14	12	36	0.78	0.72	6

SCC, squamous-cell lung carcinomas; NM, not mentioned; AUC, area under ROC curve; TP, true positive; FP, false positive; FN, false negative; TN, true negative; SEN, sensitivity; SPE, specificity; QUADAS, Quality Assessment of Diagnostic Accuracy Studies.

#### Diagnostic accuracy of miR-205 for lung cancer

**Figure 3:**
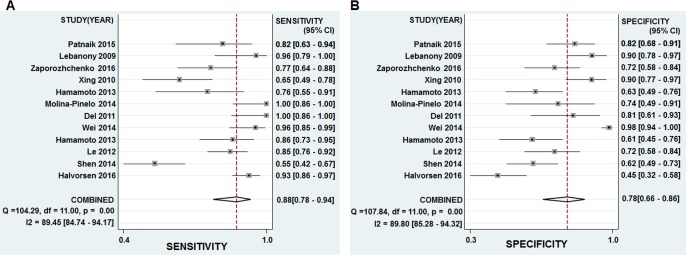
Forest plots of sensitivities and specificities from test accuracy studies of miR-205 in the diagnosis of lung cancer

**Figure 4:**
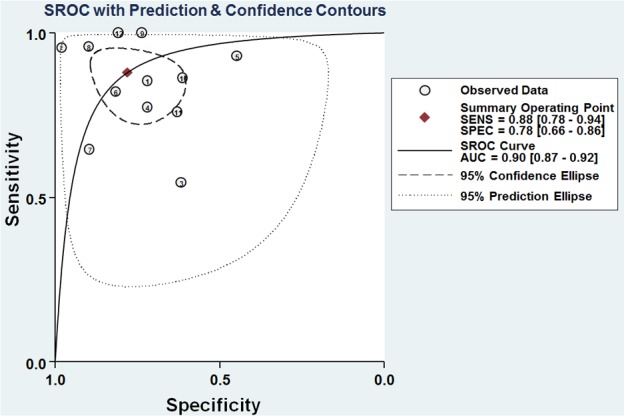
SROC curves of miR-205 for the diagnosis of lung cancer

The pooled results of sensitivity, specificity, diagnostic odds ratio (DOR), and the area under the summary receiver operator characteristic curve (AUC) were 0.88, 0.78, 25.86, and 0.90, respectively (Table 2, Figures 3 and 4), revealing that miR-205 achieved a relatively high overall accuracy for detection of lung cancer. The combined positive likelihood ratio (PLR) and negative likelihood ratio (NLR) were 4.00 and 0.16, respectively, which further suggested that miR-205 has sufficient power to confirm or exclude lung cancer (Table 2, Figure 3). Nevertheless, the *I*^2^ values of pooled sensitivity and specificity were 89.45 % and 89.80 %, respectively, indicating that there is a significant heterogeneity among the included studies.

**Table 2:** Summary results for diagnostic accuracy of miR-205 for lung cancer

Analysis	N	SEN(95% CI)	SPE(95% CI)	PLR(95% CI)	NLR(95% CI)	DOR(95% CI)	AUC(95% CI)
Ethnicity							
Asian	4	0.88(0.77-0.94)	0.81(0.52-0.94)	4.59 (1.42-14.90)	0.15(0.06-0.36)	30.43(4.16-222.26)	0.91(0.89-0.93)
non-Asian	8	0.89(0.73-0.96)	0.76(0.65-0.85)	3. 75 (2.38-5.90)	0.14(0.05-0.40)	26.56(7.19-98.17)	0.88(0.84-0.90)
Specimen							
tissue	7	0.92(0.84-0.97)	0.83(0.69-0.92)	5.56 (2.75-11.26)	0.09(0.04-0.22)	60.35(14.59-249.67)	0.95(0.93-0.97)^*^
blood	5	0.78(0.62-0.88)	0.69(0.54-0.81)	2.54 (1.72-3.76)	0.32(0.19-0.54)	8.01(3.92-16.35)	0.80(0.76-0.83)^*^
Size							
>100	5	0.85(0.69-0.94)	0.77(0.50-0.92)	3.66 (1.35-9.95)	0.19(0.07-0.51)	19.07(3.00-121.20)	0.89(0.86-0.91)
≤100	7	0.91(0.75-0.97)	0.79(0.69-0.86)	4.24 (2.78-6.49)	0.12(0.04-0.35)	35.16(9.78-126.44)	0.89(0.86-0.91)
Overall	12	0.88(0.78-0.94)	0.78(0.66-0.86)	4.00(2.47-6.49)	0.16(0.08-0.30)	25.86(9.29-71.95)	0.90(0.87-0.92)

N, number of studies; SEN, sensitivity; 95% CI, 95% confidence interval; SPE, specificity; PLR, positive likelihood ratio; NLR, negative likelihood ratio; DOR, diagnostic odds ratio; AUC, area under ROC curve; ^*^statistically significant results.

**Figure 5:**
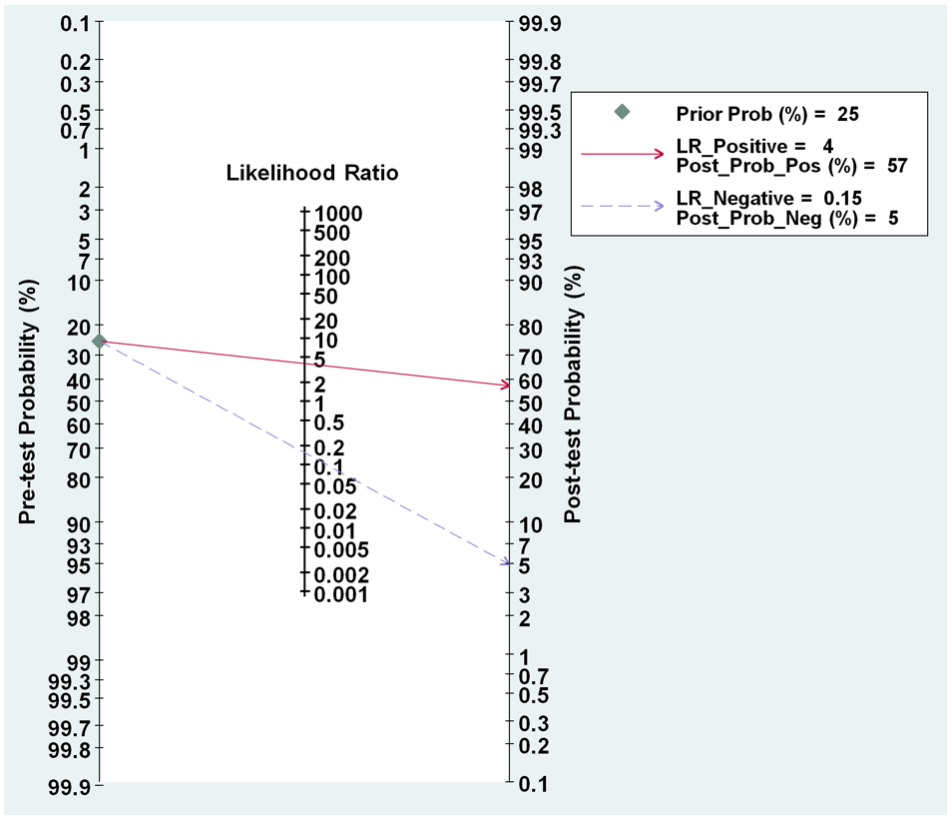
Nomogram of Fagan describes the probability miR-205 to confirm or exclude lung cancer patients

Nomogram of Fagan was used to estimate the clinical diagnostic values of miR-205 in detection of lung cancer. In details, for any people with a pre-test probability of 25 % of patients with lung cancer, positive results of miR-205 show the post-test probability of correctly diagnosing cancer would rise to 57%, while negative results of miR-205 mean the post-test probability would drop to 5 % (Figure 5). Thus, miR-205 is an important diagnostic biomarker for lung cancer.

#### Diagnostic threshold effect

The threshold effect caused by differences in the sensitivity and specificity is a major source of heterogeneity in diagnostic tests. Hence, spearman correlation coefficient of sensitivity and specificity was calculated to assess threshold effect. According to the analysis, spearman correlation coefficient in total 12 studies was 0.33, with a *P* value of 0.11 (*P* > 0.05), which indicated that the heterogeneity was not caused by the threshold effect.

#### Subgroup analysis

Subgroup analysis was performed based on ethnicity (Asian vs. Non-Asian), specimens (tissue vs. blood), and sample size (≤ 100 vs > 100) (Table 2). There was no obvious significance between Asian and non-Asian population. The subgroup analysis based on specimens indicated tissue samples have better diagnostic accuracy than blood samples for lung cancer, with sensitivity of 0.92 versus 0.78, specificity of 0.83 versus 0.69, PLR of 5.56 versus 2.54, NLR of 0.09 versus 0.32, DOR of 60.35 versus 8.01, and AUC of 0.95 versus 0.80, respectively. Meanwhile, a large sample size exhibited the similar diagnostic accuracy to studies on a small sample size.

#### Sensitivity analysis and publication bias

**Figure 6:**
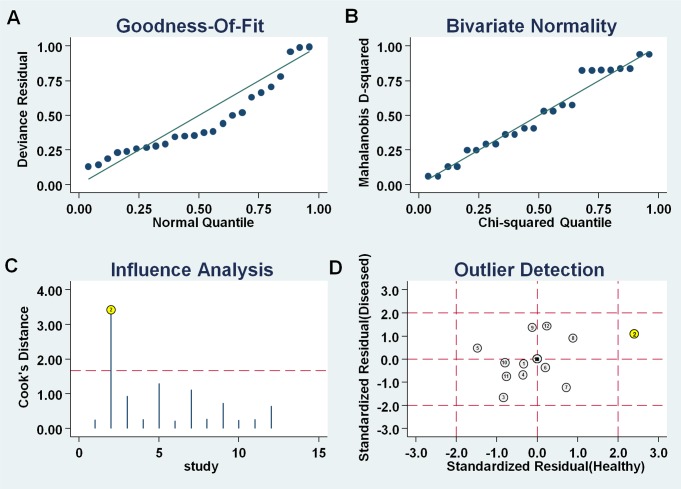
Influence analysis and outlier detection **(A)** goodness of fit, **(B)** bivariate normality, **(C)** influence analysis, and **(D)** outlier detection.

Sensitivity analysis was conducted and 1 outliner was found (Figure 6). After exclusion, the sensitivity changed from 0.88 to 0.87, specificity increased from 0.78 to 0.73, showing that there were no significant changes with our overall analysis. Combined with goodness of fit and bivariate normality analyses, we confirmed the robustness of our meta-analysis.

**Figure 7:**
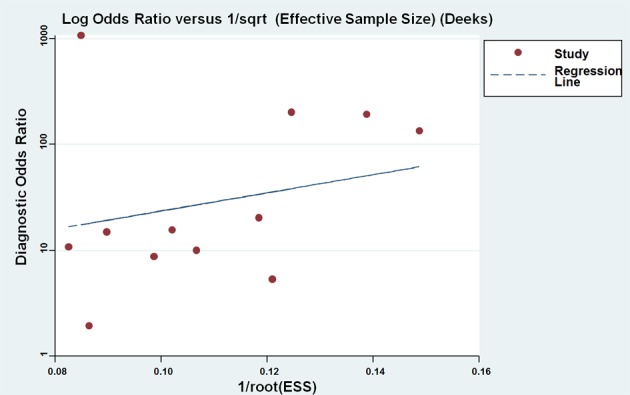
Funnel plots for the assessment of potential diagnosis bias in miR-205 assays

Deeks’ funnel plot was performed to evaluate the publication bias of the included studies. The funnel plots of diagnostic results indicated no obvious publication bias in this diagnostic meta-analysis (Figure 7). The *P* value of Deek's test was 0.54.

### Prognostic meta-analysis

#### Study characteristics and quality assessment

**Figure 8:**
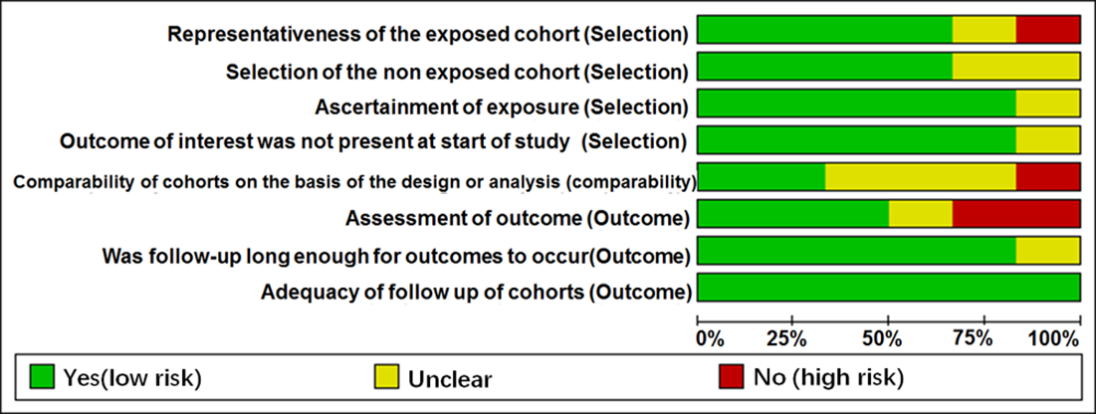
Quality assessment of prognostic accuracy for the included studies

The main features of the 6 included studies for prognosis are listed in Table 3. There were 756 participants in the 6 qualified studies. Of these studies, 2 studies investigated overall survival (OS) of lung patients, 1 reported recurrence-free survival (RFS), and 3 focused on OS as well as disease-free survival (DFS)/RFS of patients. For ethnicity, 3 studies investigated Caucasian, 2 evaluated Asian, and 1 focused on Caucasian and African. For cancer type, 4 studies focused on NSCLC, 1 focused on SCLC, and 1 focused on lung cancer. The follow-up time ranged from 9.9 months to 60 months. Five studies detected the expression of miR-205 in tissue samples except Le's study [26], which determined its expression in blood samples. The quality of the included studies was assessed by Newcastle–Ottawa Quality Assessment Scale (NOS, Supplementary Figure 2), and the quality score ranged from 5 to 7. Thus, all the included studies were regarded as moderate and high quality (Figure 8 and Table 3).

**Table 3:** Characteristics and quality assessment of prognostic clinical trials included in the meta-analysis

Author	Year	Country	Ethnicity	Cancer type	Number	Specimen	Results	Cut off	Follow-up (month)	P	HR	LL	UP	NOS
Markou	2008	Greece	Caucasian	NSCLC	48	tissue	OS	2.0	50	0.610	1.27	0.52	3.13	5
							DFS	2.0	50	0.476	1.32	0.62	2.86	5
Zhang	2012	China	Asian	NSCLC	105	tissue	OS	Mean	16.25	<0.001	42.33	1.51	148.52	6
Le	2012	China	Asian	NSCLC	82	serum	OS	Mean	30	0.689	1.23	0.45	3.37	7
							DFS	Mean	30	0.169	0.5	0.18	1.35	7
Lu	2012	USA	Caucasian	lung cancer	357	tissue	RFS	Mean	60	0.005	0.85	0.77	0.95	5
Begum	2015	USA	Caucasian/African	NSCLC	114	tissue	RFS	Mean	46.3	0.459	1.22	0.72	2.06	7
							OS	Mean	46.3	0.228	1.39	0.81	2.35	7
Mancuso	2016	Italy	Caucasian	SCLC	50	cytologic samples	OS	Median	9.9	0.226	1.46	0.79	2.69	7

SCLC, small cell lung cancer; NSCLC, non-small cell lung cancer; DFS, disease-free survival; OS, overall survival; RFS, recurrence-free survival; HR, hazard ratio; LL, lower limit of 95% confidence interval; UL, upper limit of 95% confidence interval; NOS, Newcastle–Ottawa Quality Assessment Scale.

#### Correlation between miR-205 expression and OS

**Figure 9:**
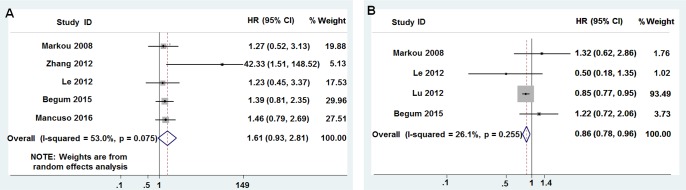
Forest plots of studies evaluating the miR-205 expression level and prognosis **(A)** Survival data are reported as overall survival (OS) and **(B)** relapse-free survival (RFS) or disease-free survival (DFS).

A total of 5 studies were used for OS analysis. Moderate heterogeneity was found among the studies on miR-205 (*P* = 0.075, I^2^= 53 %). Therefore, the pooled hazard ratio (HR) was summarized by using a random-effect model. Our results failed to demonstrate any significant association between miR-205 expression and OS (pooled HR = 1.61, 95% CI: 0.93 – 2.81, z=1.69, *P* = 0.091, Figure 9A).

#### Correlation between miR-205 expression and DFS/RFS

A total of 4 studies were used for DFS/RFS analysis. Low heterogeneity was found among the studies on miR-205 (*P*=0.255, *I*^2^=26.1%). Therefore, the pooled HR was summarized by using a fixed-effect model. Our results indicated that the increased miR-205 expression had a protective role in the prognosis of patients with lung cancer (pooled HR=0.86, 95% CI: 0.78-0.96, z=2.83, *P*=0.005, Figure 9B).

#### Publication bias

Egger's test was applied to assess the publication bias. The *P* values of Egger's test for OS and DFS/RFS were 0.144 and 0.648, respectively, suggesting no obvious publication bias exists in this meta-analysis. However, a low sensitivity will be found in the results of the Begg's test when the number of eligible studies is < 10 [38], so it was not used for this study.

## DISCUSSION

Recently, the application of miRNAs as biomarkers for cancer diagnosis and prognosis has gained much attention in recent years [39]. Accumulating evidence supports that their abnormal expression levels associated with various tumors, such as lung cancer, breast cancer, and cervical cancer [40, 41]. More importantly, miRNAs are extremely stable and present in various biological materials, like serum, plasma, and tissue. MiRNAs are also easy to be measured by means of multiple methods [42, 43]. Therefore, miRNAs can be used as reliable biomarkers for diagnosis and prognosis of cancer. In this study, we demonstrated that miR-205 is overexpressed in patients with lung cancer, which is a promising biomarker for diagnosis and prognosis of lung cancer.

### MiR-205 is a diagnostic biomarker for lung cancer

Among these tumor-specific miRNAs, miR-205 is one of the most frequently studied miRNAs [18, 23–37, 39]. Dysregulation of miR-205 was observed in many types of cancers, including lung cancer [39]. The expression of miR-205 appears to be tissue or tumor type-specific, which is useful to classify human cancers [44], distinguish tumor subtypes [18], and correlate with prognosis [24]. MiR-205 directly repressed PTEN expression and was upregulated in multiple subtypes of NSCLC [45]. MiRNA profiling of plasma fractions revealed that miR-205 levels increased in tumor-specific exosomes of patients with SCC, but its levels decreased strikingly after surgery [46]. The expression of miR-205 improved the diagnostic sensitivity for patients with SCC compared with no-SCC [18, 30, 31].

The above mentioned studies indicated that miR-205 might serve as a wonderful biomarker for diagnosis of lung cancer [18, 23, 26, 30, 35, 36], but others didn't support its roles in lung cancer detection [34, 37]. Therefore, we further evaluated the roles of miR-205 in diagnosis of lung cancer and found that miR-205 presented diagnostic sensitivity of 0.88 (95% CI = 0.78 – 0.94), specificity of 0.78 (95% CI = 0.66 – 0.86) and AUC of 0.90 (95% CI = 0.87 – 0.92). These three representative parameters confirmed the accuracy of miR-205 as a promising predictor for examining lung cancer. And our results showed that the DOR value was 25.86, which also proved that miR-205 is a useful biomarker for lung cancer detection.

MiRNA expression varies from different specimens, ethnicities, and cancer types, etc. Here, we demonstrated that miR-205 had precisely diagnostic value in lung cancer by using tissue and blood samples, especially using tissue samples. Similarly, Jiang et al [47] showed that miR-205 expression was significantly higher in NSCLC tissues and serum, which was a good diagnostic biomarker for NSCLC. As to ethnicity, He et al [48] showed that miR-205, as a critical therapeutic target, expressed differently between Chinese patients with prostate cancer and Western patients. Differently in this study, we failed to find an obvious difference between Asian population and non-Asian population.

### MiR-205 is a prognostic biomarker for lung cancer

Furthermore, miR-205 is important for the prognosis of patients with lung cancer. It has been identified as a potentially useful predictor of survival for patients with SCLC [29]. Zhang et al [25] found that high levels of miR-205 in patients with SCC lead to an increased probability of mortality, but low expression levels of miR-205 indicated the reduced short term (<10 months) mortality [37]. Lu et al [27] demonstrated that miR-205 was a great potential target for histology-specific treatment or prevention of recurrent lung cancer. The latest findings revealed that miR-205 serves as a potential biomarker for the prognosis of advanced NSCLC, and suppression of miR-205 expression decreased A549 cell biological activity by regulating Akt/mTOR/P21 signaling pathway [39]. However, some studies showed that overexpression of miR-205 had no prognostic implication for patients with NSCLC, since it was not associated with reduced DFS (or OS) and any clinic pathological feature of the patients [26, 28, 31].

To further solve the controversial results of miR-205 in prognosis of lung cancer, for the first time, we conducted this meta-analysis to evaluate the relationship between miR-205 expression and the prognosis of patients with lung cancer. Although several studies supported that increased miR-205 expression may predict a worse OS [25, 49], our results didn't support this point. On the contrary, the pooled outcome of DFS/RFS analysis in this study demonstrated that that higher miR-205 expression is a promising prognostic factor for lung cancer. Increased miR-205 expression is predictive of a better prognosis, with pooled HR of 0.86, 95% CI: 0.78-0.96, and this association was statistically significant (z = 2.83, *P* = 0.005). Our finding was supported by Zhang et al [49], who reported that miR-205 was a promising biomarker for predicting the DFS/RFS of patients with breast cancer.

### Strengths and limitations of this meta-analysis

This study had several strengths. To our knowledge, we performed a meta-analysis to evaluate the diagnostic and prognostic efficiency of miR-205 for patients with lung cancer for the first time. Moreover, different sample sources were investigated, with the aim of identifying the most suitable one for clinical application. In addition, we used comprehensive methods, including strict literature screening and quality evaluation process, to reduce bias.

Limitations do exist in the meta-analysis. Firstly, the blind design was not used in some diagnostic studies, which affects the diagnostic accuracy. Secondly, the sample size in this meta-analysis was relatively small. Thirdly, considering heterogeneity existed among these studies.

In summary, this study demonstrated that miR-205 may be used as a promising biomarker for diagnosis and prognosis for lung cancer. Our findings require further evaluation in future large-scale prospective studies.

## MATERIALS AND METHODS

### Literature search

A systematical computerized search was performed for relevant publications that investigated the roles of miR-205 in the diagnosis and prognosis of lung cancer in Embase, PubMed, Web of Science databases, and Ovid platform (up to March 31, 2017) for the terms (“lung cancer” or “lung neoplasms” or “lung tumor” or “lung tumour” or “lung malignancy” or “lung neoplasia”) and (“microRNA-205” or “miRNA-205” or “miR-205” or “hsa-miR-205”). We further manually searched the bibliographies of articles to identify the missed suitable articles.

### Literature selection

The included studies in this meta-analysis satisfied all of the following inclusion criteria: (1) patients with lung cancer; (2) the expression levels of miR-205 in blood, serum, plasma, cytologic samples, or lung cancer tissues; and (3) sufficient data on the association between miR-205 expression levels and lung cancer diagnosis or prognosis. The exclusion criteria were as follows: (1) duplicate publications; (2) review paper, case report, letter, and meta-analysis; (3) unqualified data; and (4) non-English publications.

### Data extraction

Two reviewers (Jing-Hua Li and Shan-Shan Sun) independently carefully reviewed the full text and extracted the relevant data. A third person (Ning Li) resolved the differences until all arrived at a set of similar statements. After that, the following data were extracted from each study: name of the first author, published year, country, study population characteristics (ethnicity, sample size, cancer types, specimen, follow-up time, and source of control), and relevant data for meta-analysis. For diagnostic studies, data of two-by-two tables were extracted, including false negatives (FN), true negatives (TN), true positives (TP), and false positives (FP) etc. For prognostic studies, HR of miR-205 for OS, RFS and DFS, with corresponding 95% CIs and *P* value were directly extracted from the studies, or extracted from the Kaplan-Meier curves by using a method as Tierney's study [50].

### Quality assessment

The methodological quality assessment of diagnostic study was conducted by independent team members according to the guidelines of the QUADAS-2 tool [40], which is composed of 7 item check list, and each item will be assessed as 1 score (“yes”), 0 score (“no” / “unclear”). For prognostic studies, NOS was applied to assess the quality of observational studies [51]. It consists of the following 3 parts: selection (4 items), comparability (2 items) and outcome (3 items). Thus, the quality of study was determined on a scale ranged from 0 to 9 points. Studies with seven or more points were regarded as high quality [52].

### Statistical analysis

Meta-analysis was conducted using STATA 13.0 (Stata Corporation: College Station, TX, USA) and Review Manager 5.3 (Copenhagen: Nordic Cochrane Centre, the Cochrane Collaboration, 2014) software. The bivariate meta-analysis model was employed to calculate the pooled results of sensitivity, specificity, PLR, NLR, and DOR along with their 95% CIs, and generate the summary receiver operator characteristic (SROC) curve. The AUC represents an analytical summary of test performance [53, 54]. Moreover, the amount heterogeneity among studies caused by the threshold effects was examined using spearman correlation coefficient [55]. The non-threshold effect was assessed by the Cochran’s-Q and I-squared statistics index. A low *P* value (< 0.05) for heterogeneity or high I-squared (> 50%) suggests presence of heterogeneity caused by non-threshold effect. If the heterogeneity caused by non-threshold effects existed, stratified analysis (ethnicity, specimen types, and sample size) would be used to explore the sources of heterogeneity. The Fagan's nomogram was conducted to explore the clinical diagnostic value of miR-205 in detection of cancer. The Deek's funnel plot method was used to explore publication bias, with *P* < 0.05 indicates obvious publication bias.

For the prognostic meta-analysis, HRs and their 95% CIs were used to assess the impact of miR-205 expression on survival of patients with lung cancer. Cochran’s-Q and I-squared statistics index were used to assess the heterogeneity of the pooled results. A low *P* value (< 0.05) for heterogeneity or high *I*-squared (> 50%) suggests that the random-effects model would be applied, otherwise, the fixed-effects model was used. Moreover, the Begg's test and Egger's test were used to check the publication bias. All values of the *P* < 0.05 were considered represent statistical significance.

## SUPPLEMENTARY MATERIALS FIGURES


