# Supplementary material for: MiR-205 as a promising biomarker in the diagnosis and prognosis of lung cancer
Source: Oncotarget. 2017 Aug 14;8(54):91938–49. doi: 10.18632/oncotarget.20262 (PMC5696153; doi:10.18632/oncotarget.20262)
Supplement: Supplementary file 1 [file oncotarget-08-91938-s001.pdf]

MiR-205 as a promising biomarker in the diagnosis and prognosis of lung cancer

SUPPLEMENTARY MATERIALS

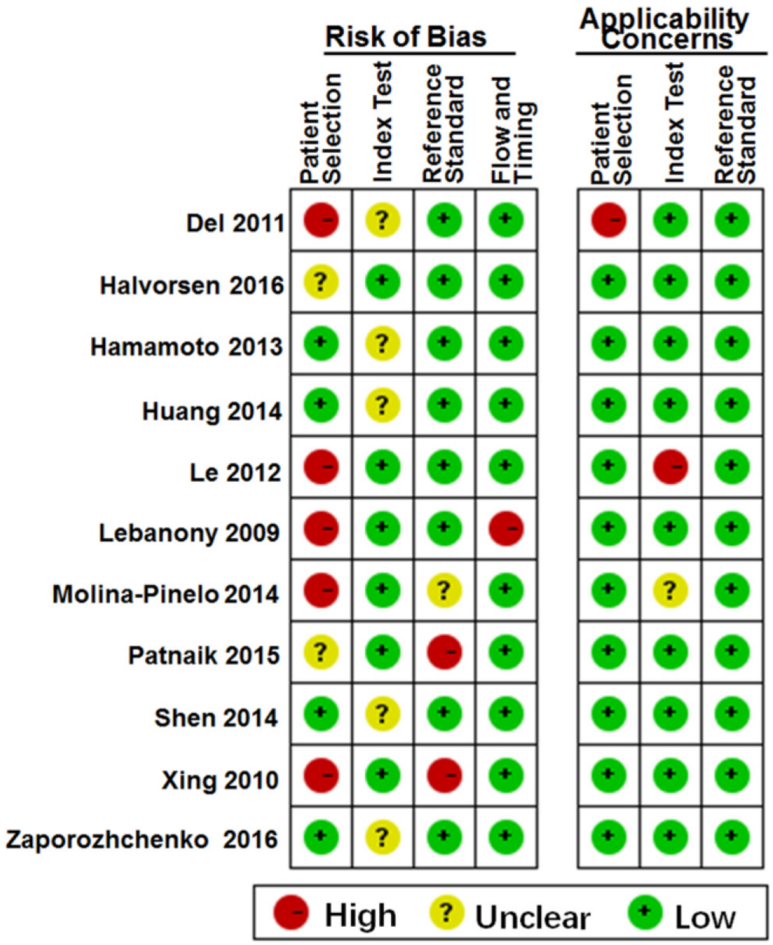

Supplementary Figure 1: Risk of bias summary regarding each domain for each included diagnostic study. Each row represents an included study. Each column represents a different evaluation criterion. Red, high risk. Yellow, medium risk. Green, low risk.

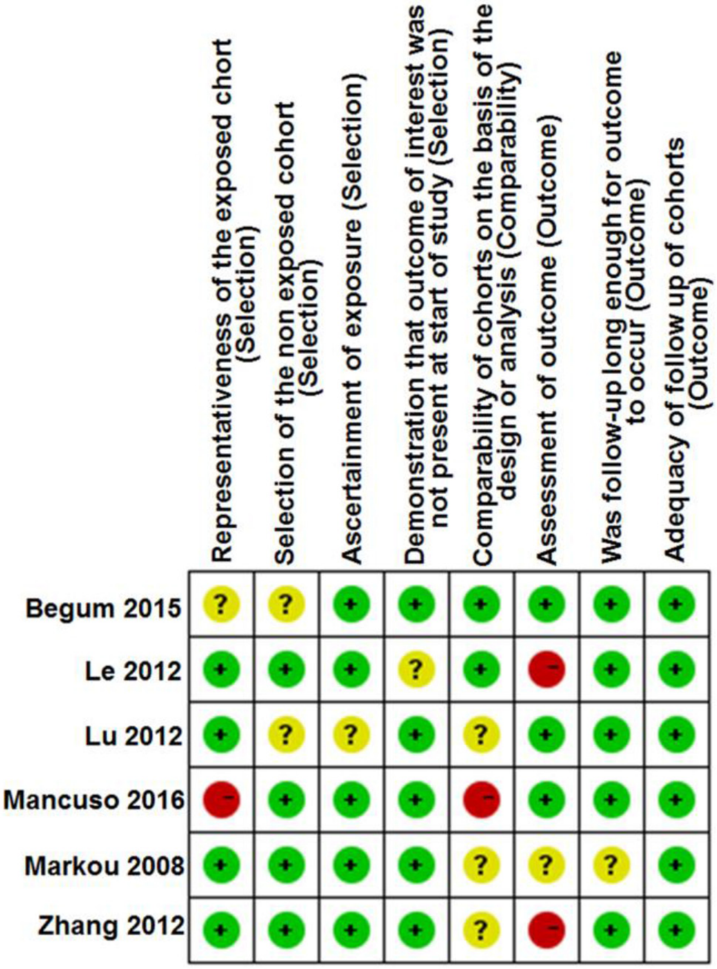

Supplementary Figure 2: Risk of bias summary regarding each domain for each included prognostic study. Each row represents an included study. Each column represents a different evaluation criterion. Red, high risk. Yellow, medium risk. Green, low risk.
